# miR-382 targeting PTEN-Akt axis promotes liver regeneration

**DOI:** 10.18632/oncotarget.6444

**Published:** 2015-12-01

**Authors:** Yihua Bei, Yang Song, Fei Wang, Jasmina Dimitrova-Shumkovska, Yang Xiang, Yingying Zhao, Jingqi Liu, Junjie Xiao, Changqing Yang

**Affiliations:** ^1^ Regeneration and Ageing Lab, Experimental Center of Life Sciences, School of Life Science, Shanghai University, Shanghai, China; ^2^ Division of Gastroenterology and Hepatology, Digestive Disease Institute, Tongji Hospital, Tongji University School of Medicine, Shanghai, China; ^3^ Department of Experimental Biochemistry and Physiology, Faculty of Natural Sciences and Mathematics, University Ss Cyril and Methodius, Skopje, Republic of Macedonia; ^4^ State Key Laboratory of Pharmaceutical Biotechnology and Department of Biochemistry, Nanjing University, Nanjing, China; ^5^ Shanghai Key Laboratory of Bio-Energy Crops, School of Life Science, Shanghai University, Shanghai, China

**Keywords:** microRNA, liver regeneration, proliferation, PTEN, Akt

## Abstract

Liver regeneration is a highly orchestrated process which can be regulated by microRNAs (miRNAs, miRs), though the mechanisms are largely unclear. This study was aimed to identify miRNAs responsible for hepatocyte proliferation during liver regeneration. Here we detected a marked elevation of miR-382 in the mouse liver at 48 hrs after partial hepatectomy (PH-48h) using microarray analysis and qRT-PCRs. miR-382 overexpression accelerated the proliferation and the G1 to S phase transition of the cell cycle both in mouse NCTC1469 and human HL7702 normal liver cells, while miR-382 downregulation had inverse effects. Moreover, miR-382 negatively regulated PTEN expression and increased Akt phosphorylation both *in vitro* and *in vivo*. Using PTEN siRNA and Akt activator/inhibitor, we further found that PTEN inhibition and Akt phosphorylation were essential for mediating the promotive effect of miR-382 in the proliferation and cell growth of hepatocytes. Collectively, our findings identify miR-382 as a promoter for hepatocyte proliferation and cell growth via targeting PTEN-Akt axis which might be a novel therapeutic target to enhance liver regeneration capability.

## INTRODUCTION

Liver possesses a tremendous regenerative capability after injury or surgical resection [1, 2]. In rodent model of 70% partial hepatectomy (PH), adult hepatocytes immediately enter G1 phase and transverse to S phase of the cell cycle with a peak of DNA synthesis at 24-40 hrs after PH, and ultimately grow to its pre-resection mass in 7-10 days [3, 4]. However, liver regenerative capability can usually be impaired under certain circumstances such as liver cirrhosis and liver failure [5, 6], highlighting the necessity to identify novel approaches to enhance liver regenerative capacity.

MicroRNAs (miRNAs, miRs), a group of small non-coding RNAs, are negative regulators of their target genes at posttranscriptional level, principally through combining to the 3′-UTR of target messenger RNA (mRNA) and reducing mRNA stability and/or translation [7-10]. Recently, miRNAs have increasingly been reported to control the process of liver regeneration [11, 12]. Among the aberrantly expressed miRNAs, miR-21 [11, 13-15], −23b [16], −122 [17], −203 [18] and −221 [19] have been shown as promoters for hepatocyte proliferation, while miR-26a [20], −33 [21], −34a [22], −127 [23], −150 [24] and −378 [11] have inverse effect. However, the mechanisms mediating the regulatory effect of miRNAs in liver regeneration are still largely unclear.

In this study, we found a significant elevation of miR-382 in the mouse liver at 48 hrs after 70% PH using microarrays and quantitative reverse transcription-polymerase chain reactions (qRT-PCRs). Our data showed that miR-382 could promote hepatocyte proliferation and cell growth *in vitro*. Additionally, miR-382 overexpression negatively correlated with PTEN expression at post transcriptional level both *in vivo* and *in vitro*. Using PTEN siRNA and Akt activator/inhibitor, we further confirmed that PTEN inhibition and Akt phosphorylation were essential for mediating the promotive effect of miR-382 in hepatocyte proliferation and cell growth. Collectively, these data indicate that miR-382 is a promoter for hepatocyte proliferation and liver regeneration via targeting PTEN-Akt axis.

## RESULTS

### miR-382 is upregulated in mouse regenerating liver

**Figure 1:**
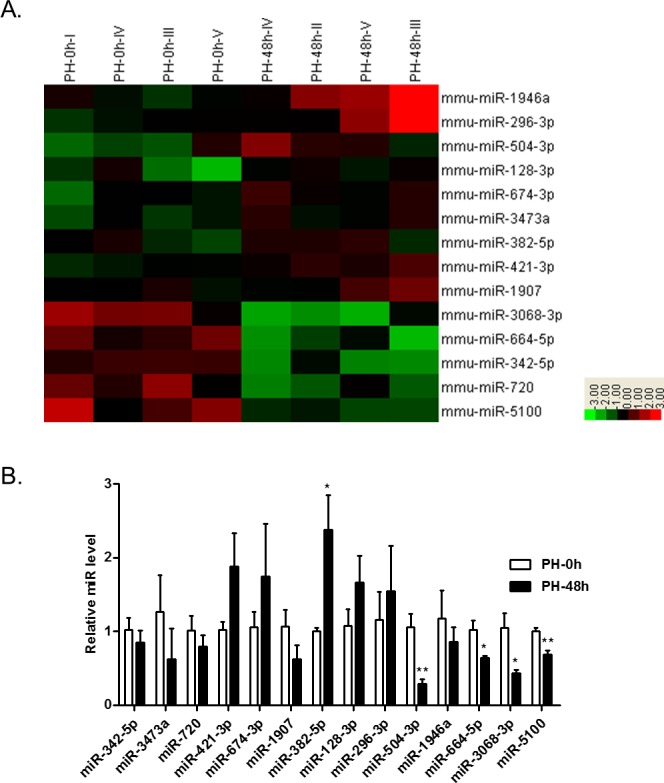
The miRNA profiles in the mouse liver at 48 hrs after PH (PH-48h) compared to those at PH-0h **A**. Heat map demonstrated the aberrantly expressed microRNAs in the mouse liver at PH-48h *vs.* those in the control liver at PH-0h (n=4). **B**. qRT-PCRs showed that miR-382-5p was upregulated, while miR-504-3p, -3068-3p, -664-5p, and -5100 were downregulated in the mouse liver at PH-48h (n=5). *, *P*<0.05; **, *P*<0.01.

Our group has previously demonstrated that hepatocyte proliferation peaked at 48 hrs after PH in the mouse liver as represented by a peak of PCNA protein level and EdU positive cells [4]. In the current study, the microarray analysis was used to evaluate miRNA profiles in the mouse liver during the proliferative phase of liver regeneration (PH-48h) in comparison to those in the control liver (PH-0h). As shown in the heat map (Figure 1A), miR-1946a, -296-3p, -504-3p, -128-3p, -674-3p, -421-3p, -3473a, -1907, and -382-5p were found to be upregulated, while miR-3068-3p, -664-5p, -342-5p, -5100, and -720 downregulated in the mouse liver at 48 hrs post PH (for data see Table 1). Next, qRT-PCRs were further conducted, verifying that the expression of miR-382-5p was significantly increased, while miR-3068-3p, -664-5p, and -5100 decreased in the mouse liver at PH-48h (Figure 1B). As miR-504-3p was found to be downregulated in the mouse liver at PH-48h using qRT-PCR which was contrary to the result of microarrays (Figure 1A and 1B), miR-504-3p was then excluded for subsequent functional assays. Among the aberrantly expressed miRNAs, miR-3068-3p, -664-5p, and -5100 did not impact the proliferation of mouse NCTC1469 liver cells *in vitro* as measured by EdU incorporation assay (data not shown). Noteworthy, miR-382-5p (previous IDs: miR-382) overexpression or suppression was found to be effective to modify hepatocyte proliferation (for detail see following results). Thus, miR-382 has been screened out for further investigation regarding its role in hepatocyte proliferation and liver regeneration in the present study.

**Table 1:** Microarray analysis showing dysregulated miRNAs in the mouse liver at 48 hrs after PH (PH-48h) versus those in the control liver at PH-0h (n=4)

Systematic Name	*P* value	Fold-change	Regulation
mmu-miR-1946a	0.014	5.238	up
mmu-miR-3068-3p	0.013	4.103	down
mmu-miR-296-3p	0.042	3.958	up
mmu-miR-504-3p	0.025	3.299	up
mmu-miR-128-3p	0.036	2.881	up
mmu-miR-664-5p	0.037	2.656	down
mmu-miR-674-3p	0.022	2.361	up
mmu-miR-342-5p	0.031	2.358	down
mmu-miR-421-3p	0.003	2.355	up
mmu-miR-3473a	0.013	2.279	up
mmu-miR-5100	0.044	2.240	down
mmu-miR-720	0.036	2.185	down
mmu-miR-1907	0.023	2.071	up
mmu-miR-382-5p	0.030	2.001	up

### miR-382 promotes hepatocyte proliferation and cell growth *in vitro*

**Figure 2:**
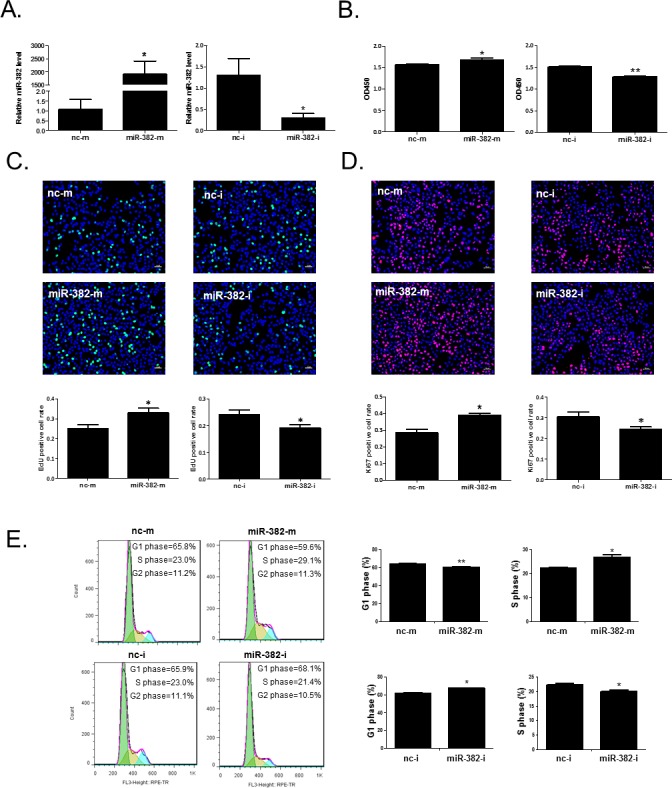
miR-382 promotes the proliferation and cell growth of mouse NCTC1469 liver cells **A.** qRT-PCR analysis for miR-382 level in NCTC1469 cells transfected with miR-382 mimics (miR-382-m), miR-382 inhibitor (miR-382-i), or their respective negative controls (nc-m or nc-i) (n=5). CCK-8 cell counting (n=10) **B.**, EdU (green) cell proliferation assay (n=5) **C.**, and Ki67 (red) immunostaining (n=5) **D.** demonstrated that miR-382 mimics promoted while miR-382 inhibitor reduced NCTC1469 cell proliferation. Nuclei were counterstained with Hoechst (blue). Original magnification 100x, scale bar = 50 μm. **E.** Flow cytometry showed that miR-382 mimics induced a G1 to S phase transition of the cell cycle of NCTC1469 cells, while miR-382 inhibitor had inverse effect (n=5). *, *P*<0.05; **, *P*<0.01.

**Figure 3:**
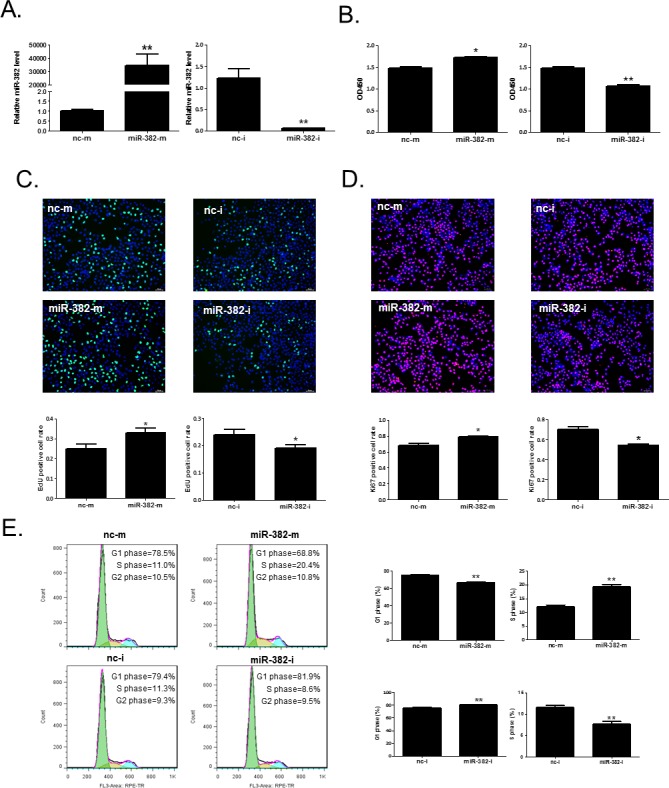
miR-382 promotes the proliferation and cell growth of human HL7702 liver cells **A.** qRT-PCR analysis for miR-382 level in HL7702 cells transfected with miR-382 mimics (miR-382-m), miR-382 inhibitor (miR-382-i), or their respective negative controls (nc-m or nc-i) (n=5). CCK-8 cell counting (n=10) **B.**, EdU (green) cell proliferation assay (n=5) **C.**, and Ki67 (red) immunostaining (n=5) **D.** demonstrated that miR-382 mimics promoted while miR-382 inhibitor reduced HL7702 cell proliferation. Nuclei were counterstained with Hoechst (blue). Original magnification 100x, scale bar = 50 μm. **E.** Flow cytometry showed that miR-382 mimics induced a G1 to S phase transition of the cell cycle of HL7702 cells, while miR-382 inhibitor had inverse effect (n=5). *, *P*<0.05; **, *P*<0.01.

To investigate the effects of miR-382 on the cell growth and proliferation of hepatocytes, miR-382 mimics, inhibitor, or their negative controls were transfected to mouse NCTC1469 and human HL7702 normal liver cells, respectively. MiR-382 mimics increased, while miR-382 inhibitor reduced miR-382 level in NCTC1469 and HL7702 liver cells, confirming that these mimics and inhibitor took effects (Figures 2A and 3A). CCK-8 cell counting, EdU cell proliferation assay, and Ki67 immunostaining showed that miR-382 mimics promoted, while miR-382 inhibitor reduced the proliferation of NCTC1469 liver cells (Figure 2B-2D). We further found that miR-382 overexpression was associated with a reduced cell population in G1 phase and an increased cell population in S phase using flow cytometry, indicating that miR-382 promoted the transition of NCTC1469 cell population from G1 phase to S phase of the cell cycle, while miR-382 downregulation had inverse effect (Figure 2E). Meanwhile, the promotive effect of miR-382 in the proliferation and cell growth of hepatocytes were also found in human HL7702 liver cells (Figure 3B-3E). Thus, miR-382 is validated as a promoter for cell growth and proliferation of hepatocytes *in vitro*.

### miR-382 overexpression negatively correlates with PTEN protein level and parallels with increased Akt phosphorylation both *in vitro* and *in vivo*

**Figure 4:**
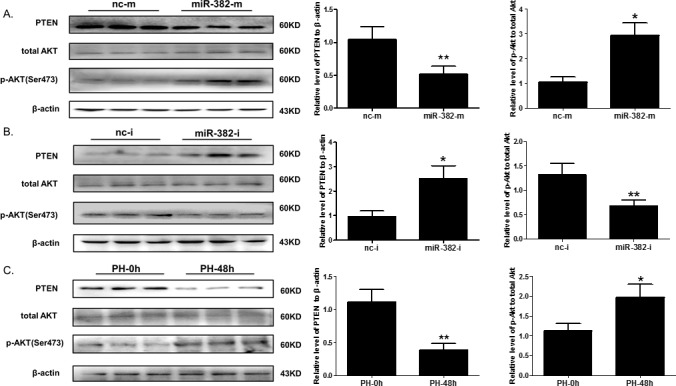
miR-382 negatively correlates with PTEN expression at protein level both *in vitro* and *in vivo* **A.** Western blot analysis for PTEN, p-AKT, and total AKT in NCTC1469 cells transfected with miR-382 mimics (miR-382-m) or negative control (nc-m) (n=3). **B.** Western blot analysis for PTEN, p-AKT, and total AKT in NCTC1469 cells transfected with miR-382 inhibitor (miR-382-i) or negative control (nc-i) (n=3). **C.** Western blot analysis for PTEN, p-AKT, and total AKT in the mouse liver at 48 hrs after PH (PH-48h) compared with control mouse liver (PH-0h) (n=3). β-actin was used as loading control. *, *P*<0.05; **, *P*<0.01.

PTEN has previously been confirmed as a direct target of miR-382 in HIF-1α-stimulated vascular endothelial cells [25]. Here we demonstrated that miR-382 mimics reduced, while miR-382 inhibitor increased PTEN expression at protein level in NCTC1469 liver cells (Figure 4A and 4B). It is well known that Akt phosphorylation is negatively regulated by PTEN, which contributes to the cell growth and proliferation [26, 27]. We further found that miR-382 overexpression increased Akt phosphorylation in NCTC1469 cells, while miR-382 downregulation showed inverse effect (Figure 4A and 4B). The total Akt protein level was found unchanged in NCTC1469 cells either with miR-382 mimics or inhibitor transfection (Figure 4A and 4B). Intriguingly, PTEN expression was also found to be reduced, while Akt phosphorylation was enhanced in the mouse liver at PH-48h compared to control mouse liver (PH-0h), though total Akt was not altered (Figure 4C). These data indicate a potential relationship between miR-382 overexpression and the PTEN/Akt signaling pathway during the proliferative phase of liver regeneration that might contribute to the cell growth and proliferation of hepatocytes.

### miR-382 promotes hepatocyte proliferation and cell growth via targeting PTEN

**Figure 5:**
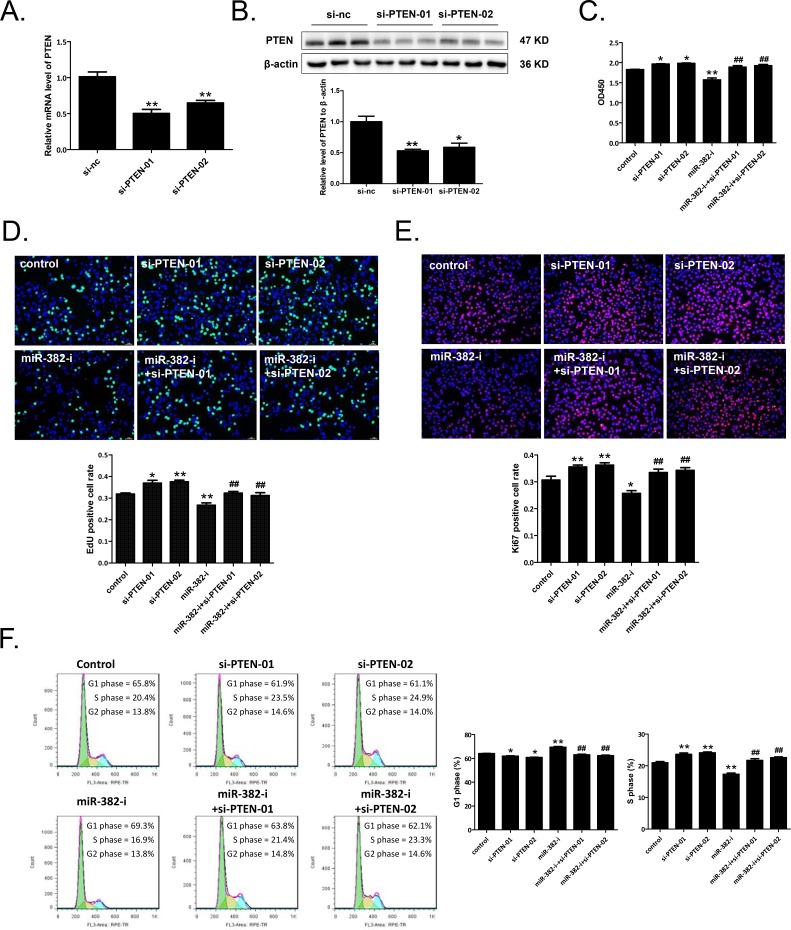
siRNA-PTEN reverses miR-382 inhibition-induced NCTC1469 cell growth arrest siRNA-PTEN (sequence-01 or -02) reduced PTEN mRNA **A.** and protein **B.** levels in NCTC1469 cells as measured by qRT-PCRs (n=5) and Western blot analysis (n=3). CCK-8 cell counting (n=10) **C.**, EdU (green) cell proliferation assay (n=5) **D.**, and Ki67 (red) immunostaining (n=5) **E.** demonstrated that siRNA-PTEN (sequence-01 or -02) reversed the suppressive effect of miR-382 inhibitor (miR-382-i) on the proliferation of NCTC1469 cells. Nuclei were counterstained with Hoechst (blue). Original magnification 100x, scale bar = 50 μm. **F.** Flow cytometry showed that miR-382 inhibitor-induced G1 phase arrest of NCTC1469 cells was significantly abolished by siRNA-PTEN (sequence-01 or -02) (n=5). *, *P*<0.05 *vs.* control; **, *P*<0.01 *vs.* control; #, *P*<0.05 *vs.* miR-382-i; ##, *P*<0.01 *vs.* miR-382-i.

Using siRNA-PTEN (sequence-01 or -02), the PTEN mRNA and protein levels were significantly reduced in NCTC1469 liver cells (Figure 5A and 5B). CCK-8 cell counting, EdU cell proliferation assay, and Ki67 immunostaining showed that NCTC1469 cell proliferation was reduced by miR-382 inhibitor, while increased by siRNA-PTEN-01 or -02 (Figure 5C-5E). Meanwhile, miR-382 inhibitor caused a G1 phase arrest in NCTC1469 cells, while siRNA-PTEN-01 or -02 induced a transition of the cell population from G1 to S phase (Figure 5F). More importantly, co-transfection with miR-382 inhibitor and siRNA-PTEN (sequence-01 or -02) abolished the inhibitory effect of miR-382 inhibitor on hepatocyte proliferation and cell growth (Figure 5C-5F).

**Figure 6:**
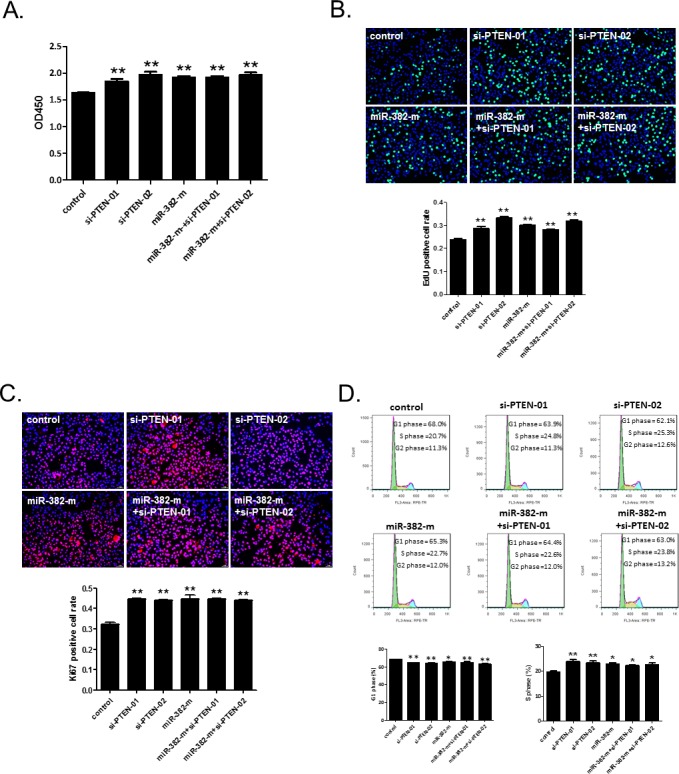
siRNA-PTEN does not further enhance the promotive effect of miR-382 on the proliferation and cell growth of NCTC1469 cells CCK-8 cell counting (n=10) **A.**, EdU (green) cell proliferation assay (n=5) **B.**, and Ki67 (red) immunostaining (n=5) **C.** demonstrated that co-transfection with miR-382 mimics (miR-382-m) and siRNA-PTEN (sequence-01 or -02) did not show an additional effect on the proliferation of NCTC1469 cells as compared to those transfected with miR-382-mimics or siRNA-PTEN alone. Nuclei were counterstained with Hoechst (blue). Original magnification 100x, scale bar = 50 μm. **D.** Flow cytometry showed that siRNA-PTEN (sequence-01 or -02) did not further enhance the miR-382 mimics-induced G1 to S phase transition of the cell cycle of NCTC1469 cells (n=5). *, *P*<0.05 *vs.* control; **, *P*<0.01 *vs.* control.

To further examine to which extent PTEN modulation mediates the role of miR-382 on hepatocyte proliferation, miR-382 mimics and siRNA-PTEN were co-transfected to NCTC1469 liver cells. Using CCK-8 cell counting, EdU cell proliferation assay, and Ki67 immunostaining, we found that either miR-382 mimics or siRNA-PTEN (sequence-01 or -02) promoted hepatocyte proliferation (Figure 6A-6C). Transfection with miR-382 mimics or siRNA-PTEN (sequence-01 or -02) alone also induced a transition of cell population from G1 to S phase in NCTC1469 cells (Figure 6D). However, co-transfection with miR-382 mimics and siRNA-PTEN did not further enhance the proliferation or cell growth of hepatocytes (Figure 6A-6D). These data suggest that the promotive effect of miR-382 on hepatocyte proliferation and cell growth is closely related to PTEN inhibition.

### Akt activation is required for miR-382 overexpression-induced hepatocyte proliferation

**Figure 7:**
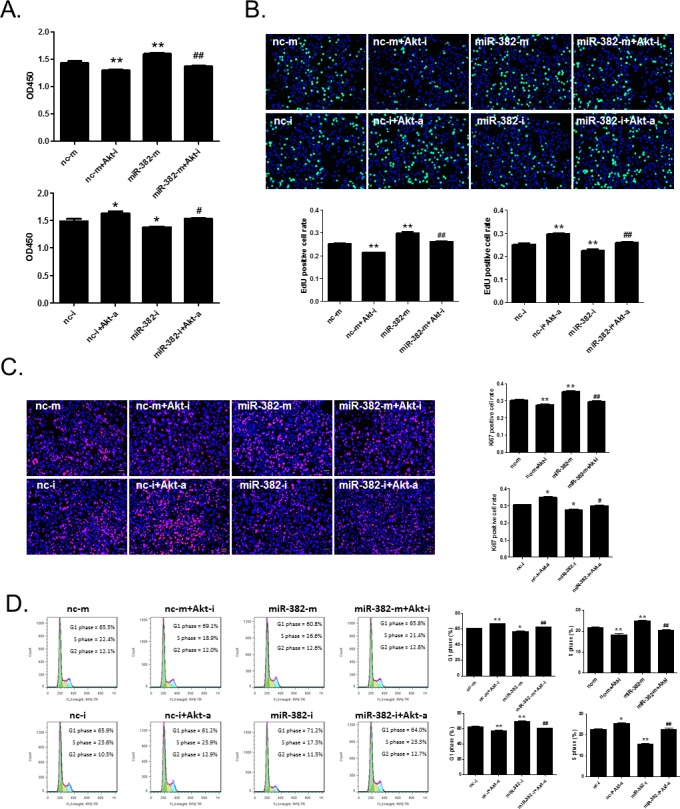
Akt activation is involved in miR-382 overexpression-induced hepatocyte proliferation CCK-8 cell counting (n=10) **A.**, EdU (green) cell proliferation assay (n=5) **B.**, Ki67 (red) immunostaining (n=5) **C.**, and flow cytometry (n=5) **D.** demonstrated that Akt activator (Akt-a) reversed miR-382 inhibitor (miR-382-i)-induced suppression of NCTC1469 cell proliferation and cell cycle progression, while Akt inhibitor (Akt-i) abolished miR-382 mimics (miR-382-m)-induced cell proliferation and cell growth. Nuclei were counterstained with Hoechst (blue). Original magnification 100x, scale bar = 50 μm. *, *P*<0.05 *vs*. control; **, *P*<0.01 *vs.* control; #, *P*<0.05 *vs*. miR-382-m or miR-382-i; ##, *P*<0.01 *vs*. miR-382-m or miR-382-i.

As PTEN is an inhibitor for Akt phosphorylation, we further investigated whether Akt contributes to the promotive effect of miR-382 on hepatocyte proliferation and cell growth. Here we showed that miR-382 mimics or Akt activator enhanced, while miR-382 inhibitor or Akt inhibitor reduced the proliferation of NCTC1469 liver cells (Figure 7A-7C). Meanwhile, miR-382 mimics or Akt activator induced a G1 to S phase transition of NCTC1469 cells, while miR-382 inhibitor or Akt inhibitor caused a G1 phase arrest (Figure 7D). Noteworthy, co-treatment with miR-382 mimics and Akt inhibitor significantly abolished miR-382 overexpression-induced hepatocyte proliferation and cell growth (Figure 7A-7D). Also, Akt activator could reverse miR-382 inhibition-induced NCTC1469 cell growth arrest (Figure 7A-7D). These data, together with the results of PTEN, fully support that the PTEN/Akt signaling pathway is a downstream mechanism mediating the role of miR-382 in hepatocyte proliferation and cell growth.

## DISCUSSION

Liver regeneration after PH is principally mediated by the proliferation of hepatocytes which can be impacted by dysregulated miRNAs, though the underlying molecular mechanisms are still largely unclear. In the current study, we found a marked induction of miR-382 in the mouse liver at 48 hrs after 70% PH. miR-382 overexpression promotes hepatocyte proliferation and G1/S phase transition of the cell cycle *in vitro*. Moreover, miR-382 negatively regulates PTEN expression and increases Akt phosphorylation both *in vitro* and *in vivo*. We further confirm that PTEN inhibition and Akt phosphorylation are required for miR-382 overexpression-induced hepatocyte proliferation and cell cycle progression. Thus, the present study identifies miR-382 as a promoter for hepatocyte proliferation and cell growth via targeting PTEN-Akt axis.

We previously established the mouse model of PH demonstrating that hepatocyte proliferation peaked at 48 hrs after PH as represented by a peak of PCNA protein level and EdU-positive cells [4]. In the current study, we particularly focused on the roles of miRNAs in the proliferative phase of liver regeneration. Thus, miRNA array and qRT-PCRs were performed to identify dysregulated miRNAs in liver tissues at 48 hrs post hepatectomy. The upregulation of miR-382 during the proliferative phase of liver regeneration is intriguing. miR-382, one of the miRNA located in chromosome 14q32 locus, has been reported to be involved in angiogenesis as well as in cancer growth and invasion [25, 28-31]. Previously, miR-382 induced by HIF-1α has been identified as an angiogenic miRNA in vascular endothelial cells [25]. Besides that, increased serum miR-382 has been suggested to be a potential biomarker for breast cancer [32]. On the other hand, miR-382 has been identified as metastasis-suppressive miRNA in melanoma [33]. Also, miR-382 inhibits tumor growth and metastasis in osteosarcoma [34, 35]. Thus, the biological functions of miR-382 are tissue and cell dependent. In the present study, we found a marked elevation of miR-382 in mouse liver at PH-48h, which prompted us to further investigate the role of miR-382 in liver regeneration.

Our data show that miR-382 overexpression increases cell proliferation and induces a G1 to S phase transition of the cell cycle in both mouse NCTC1469 and human HL7702 liver cells, while miR-382 inhibition exhibits inverse effects, indicating miR-382 as a promoter for hepatocyte proliferation and cell cycle progression that might contribute to liver regeneration at proliferative stage. Previous studies have reported that miRNAs could impact hepatocyte proliferation and cell cycle progression via regulating cyclins and cyclin-dependent kinases (CDK). For example, miR-21 and miR-221 are responsible for cyclin D1 induction, leading to a rapid G1 to S phase transition of the cell cycle of hepatocytes during liver regeneration [14, 19]. On the contrary, miR-33 could inhibit cyclin D1 and CDK6 at mRNA level, thus causing reduced cell proliferation and cell cycle progression during liver regeneration [21]. As certain cyclins such as cyclin D1 and cyclin E are key regulators for G1/S phase transition, further study is needed to examine the molecular mechanisms by which miR-382 impacts the proliferation and cell cycle progression of hepatocytes [36-39].

PTEN-regulated Akt activation is a key signaling pathway which controls cell growth, survival, and proliferation [40-42]. PTEN, has previously been identified as a direct target gene of miR-382 contributing to hypoxia-induced angiogenesis [25]. Also, PTEN has been shown to be inversely regulated by miR-21, and the latter promotes hepatocyte proliferation *in vitro* [13]. However, whether the PTEN/Akt axis is regulated by miR-382 in hepatocytes is unknown. Our data show that PTEN protein level is downregulated, while Akt phosphorylation is enhanced in the mouse liver at 48 hrs after PH. Furthermore, we demonstrate that miR-382 negatively regulates PTEN expression and increases Akt phosphorylation in cultivated hepatocytes. Using PTEN siRNA and Akt activator/inhibitor, our data further provide key evidence indicating that Akt phosphorylation, at least in part associated with PTEN inhibition, is essential for miR-382 overexpression-induced hepatocyte proliferation and cell growth.

Several limitations of this study need to be highlighted. First, as multiple secreted and soluble factors, such as tumor necrosis factor (TNF), interleukin-6 (IL-6), hepatocyte growth factor (HGF), epidermal growth factor (EGF) and transforming growth factor-α (TGF-α), are responsible for initiating and promoting the liver regeneration process [43], it will be of interest to examine whether miR-382 upregulation during liver regeneration is related to these factors. Second, as we know, *in vitro* normal hepatocytes are already primed for mitosis which is sensitive for growth factors like HGF, however hepatocytes *in vivo* has a low sensitivity to these factors unless they are primed or activated by TNF and IL-6 [44, 45]. Indeed, it will be highly needed to further examine the *in vivo* effect of miR-382 in liver regeneration in the future. Finally, non-parenchymal cells as well as oval/progenitor cells also contribute to liver regeneration [46, 47]. Whether miR-382 regulates newborn hepatocytes generated from liver stem cells remains a topic for further investigation.

In conclusion, the present study shows an induction of miR-382 in the mouse liver during the proliferative phase of liver regeneration, and further demonstrates that miR-382 overexpression promotes hepatocyte proliferation and cell growth via targeting PTEN-Akt axis. The overexpression of miR-382 may be considered as a prospective novel therapeutic target to improve liver regeneration.

## MATERIALS AND METHODS

### Mouse model of partial hepatectomy (PH)

Eight-week-old pathogen-free male C57BL/6 mice were purchased from Shanghai Laboratory Animal Center (SLAC). 70% PH was conducted as previously described [4]. Briefly, mice were anaesthetized with intraperitoneal injection of 1% pentobarbital sodium (50 mg/kg), followed by abdominal median incision and hepatectomy of the median and left lobes of the liver. After the liver was resected, the abdominal incision was closed and mice were maintained in 37°C environment for anesthesia recovery. At 48 hrs after PH (PH-48h), mice were sacrificed and the livers were harvested and immediately kept into liquid azote. The liver tissues were then conserved at −80°C until RNA or protein extraction. The control mice received the same 70% PH but sacrificed at 0 hr after PH (PH-0h). This study was approved by the local ethical committees and all animal experiments were conducted under the guidelines on humane use and care of laboratory animals for biomedical research published by National Institutes of Health (No. 85-23, revised 1996).

### miRNA microarray analysis

Total RNAs were isolated from liver tissues and quantified by the NanoDrop ND-2100 (Thermo Scientific). After the control of RNA integrity using Agilent 2100 (Agilent Technologies), total RNAs were tailed with Poly A, labeled with Biotin, and then hybridized for 16 hrs at 48°C on Affemetrix miRNA 3.0 Array. GeneChips were washed and stained in the Affymetrix Fluidics Station 450. The arrays were scanned by the Affymetrix Scanner 3000 (Affymetrix) and the array images were analyzed using Affymetrix GeneChip Command Console 4.0 software (Affymetrix) to get raw data and then provide RMA normalization. Using Genespring 12.5 software (Agilent Technologies), the probes that at least 75% of samples in any 1 condition out of 2 conditions have flags in “P” were chosen for further data analysis. The differentially expressed miRNAs, with a fold change>= 2.0 and a P value < 0.05 between the groups PH-0h and PH-48h, were chosen for further validation using qRT-PCRs. The MIAME-compliant data have been submitted to Gene Expression Omnibus (GEO, platform ID: GSE68451).

### Cell culture and treatment

Mouse NCTC1469 normal liver cells were maintained in Dulbecco's Modified Eagle's Medium (Hyclone, USA) supplemented with 10% fetal bovine serum (Hyclone, USA) and 1% penicillin-streptomycin (Keygen, China) at 37°C in 5% CO_2_ environment. Human HL7702 normal liver cells were maintained in RPMI-1640 medium (Hyclone, USA) supplemented with 20% fetal bovine serum (Hyclone, USA) and 1% penicillin-streptomycin (Keygen, China) at 37°C in 5% CO_2_ environment.

For overexpression or suppression of miR-382, cells were transfected with miR-382 mimics (50 nM, RiboBio, China), miR-382 inhibitor (100 nM, Ribobio, China), or their negative controls for 48 hrs, respectively. To further investigate the role of PTEN and Akt in miR-382-associated hepatocyte proliferation and cell growth, cells were transfected with siRNA-PTEN (sequence-01 or 02, 75 nM, Ribobio, China) or siRNA-negative control for 48 hrs, or treated with Akt activator SC 79 (4 μg/mL, No. 4635, Tocris, UK) or Akt inhibitor LY 294002 (25 μM, No. 1130, Tocris, UK) for 24 hrs, respectively.

### Cell counting kit (CCK-8) for cell proliferation analysis

A proliferation assay was performed using CCK-8 (Dojindo, Japan) according to the manufacturer's instructions. Briefly, NCTC 1469 or HL7702 cells were seeded in 96-well plates at a density of 2×10^5^/ml. After 48 hrs of treatment, CCK-8 solution was added to each well and incubated for 30 min at 37°C. The absorbance was measured at 450 nm using a spectrophotometer.

### EdU incorporation assay

To detect the DNA synthesis of hepatocytes, NCTC 1469 or HL7702 cells were seeded in 96-well plates at a density of 1×10^5^/ml. 50 μM EdU was added into the medium and incubated for 2 hrs before the end of 48 hrs of cell treatment. After cells were washed 3 times with PBS and fixed with 4% paraformaldehyde (PFA) for 30 min, EdU staining was conducted using Cell-Light™ EdU Apollo®488 *In Vivo* Imaging Kit (RiboBio, China). Nuclei were counterstained with Hoechst (Sigma, USA). Digital images were acquired under fluorescence microscopy (Leica, Germany) with original magnification of 100x. The results were presented as EdU positive cell rate which was analyzed with ImageJ software.

### Immunostaining for Ki67

To determine the proliferation of hepatocytes, NCTC 1469 or HL7702 cells were seeded in 96-well plates at a density of 1×10^5^/ml. After 48 h of treatment, cells were washed 3 times with PBS and fixed with 4% PFA for 30 min. Subsequently, cells were washed again with PBS for 3 times and permeabilized with 0.5% Triton X-100 for 20 min. Next, cells were pretreated with 5% BSA in PBS for 1 hr and incubated with rabbit anti-Ki67 antibody (1:100, ab15580, Abcam, USA) overnight at 4°C. Then cells were incubated with Rhodamine-conjugated goat anti-rabbit secondary antibody (1:200, Keygen, China) for 1 hr. Finally, nuclei were counterstained with Hoechst (Sigma, USA). Digital images were acquired under fluorescence microscopy (Leica, Germany) with original magnification of 100x. The results were presented as Ki67 positive cell rate which was analyzed with ImageJ software.

### Flow cytometry for cell cycle analysis

NCTC 1469 or HL7702 cells were seeded in 12-well plates at a density of 2×10^5^/ml. After 48 hrs of treatment, cells were detached with trypsin, washed 3 times with ice cold PBS, and fixed in 70% ethanol at −20°C overnight. Cells were then suspended in a solution containing 0.5 mg/L propidium iodide (PI) and ribonuclease A. Cellular DNA content was analyzed using MoFlo XDP Cell Sorter (Beckman Coulter). The results were presented as the percentage of cell population in each phase of the cell cycle which was determined using FlowJo 7.6 software (Treestar Inc., USA).

### RNA extraction and qRT-PCRs

Total RNAs were isolated using the RNAiso Reagent extraction kit (Takara, Japan). For mRNA detection, a total of 400 ng RNA was subjected to reverse transcription-PCR reaction using iScript™ cDNA Synthesis Kit (Bio-Rad, USA). qRT-PCR was performed in triplication using SYBR Green PCR Master Mix (BioRad, USA) and CFX96 Touch™ Real-Time PCR Detection System (BioRad, USA) with the following cycle parameters: 95°C 3 min, (95°C 15 s, 60°C 30 s, 72°C 30s) for 40 cycles. The primer sequences (forward and reverse) are as follows: PTEN: TGGATTCGACTTAGACTTGACCT, GCGGTGTCATAATGTCTCTCAG; β-actin: GGCTGTATTCCCCTCCATCG, CCAGTTGGTAACAATGCCATGT. For miRNA detection, a total of 400 ng RNA was subjected to reverse transcription-PCR with Bulge-Loop™ miRNA qRT-PCR Primer Set (RiboBio, China) and CFX96 Touch™ Real-Time PCR Detection System (BioRad, USA). The following cycle parameters were used: 95°C 20 s, (95°C 10 s, 60°C 20 s, 72°C 10s) for 40 cycles. β-actin or U6 was used as internal control to equal cDNA content. The fold change of each gene expression was calculated using the 2^−ΔΔCT^ method.

### Western blot analysis

The total protein extracts of liver cells or liver tissues were prepared for Western blot analysis. Briefly, cells or liver tissues were lysed in RIPA lysis buffer (Beyotime, China) supplemented with 1 mM PMSF (Beyotime, China). A total of 30 μg of protein was subjected to electrophorese on 12% SDS-PAGE gels, transferred to PVDF membranes, and incubated with primary antibodies against Akt (1:1000, AP0059, Bioworld, USA), phospho-Akt Ser^473^ (1:1000, BS4007, Bioworld, USA) and PTEN (1:1000, ab32199, Abcam, USA) at 4°C overnight. Blots were then incubated with corresponding HRP-conjugated secondary antibodies for 1 hr. The protein levels were detected using enhanced chemiluminescence (ECL) system (Pierce Biotechnology Inc., USA) and analyzed with Image Lab software (Bio-Rad). β-actin (1:1000, AP0060, Bioworld, USA) was used as loading control.

### Statistical analysis

Data are presented as the mean ± standard error of mean (SEM). Statistical analysis was performed using independent student T-test or ANOVA with post hoc tests. P value of <0.05 was accepted as statistically significant. Statistical analysis was carried out with SPSS 20.0 software.
